# Conserved rod-to-spherical shape transitions in *Escherichia coli* through primordial experimental adaptations

**DOI:** 10.1042/BSR20260045

**Published:** 2026-05-14

**Authors:** Hui Lu, Yueyue Zhang, Boying Xu, Di Tian, Yang Xia, Tetsuya Yomo, Jian Xu

**Affiliations:** 1 State Key Laboratory of Plant Trait Design, Center for Excellence in Molecular Plant Sciences, Chinese Academy of Sciences, Shanghai 200032, China; 2 Laboratory of Biology and Information Science, School of Life Sciences, East China Normal University, Shanghai 200062, China; 3 School of Ecological and Environmental Sciences, East China Normal University, Shanghai, 200241, China; 4 Laboratory of Insect Genome Science, Kyushu University Graduate School of Bioresource and Bioenvironmental Sciences, Motooka 744, Nishi-ku, Fukuoka 819-0395, Japan

**Keywords:** Bacterial morphology, Experimental evolution, Genomic mutations, Oleic acid vesicle, Primordial environment

## Abstract

Cell shape plays a fundamental role in bacterial physiology, yet the evolutionary stability of morphological adaptations remains poorly understood. Our previous work suggested that *Escherichia coli* can evolve a spherical form under primordial-like conditions. Here, we extended experimental evolution for approximately 1000 generations in oleic acid vesicle (OAV)-supplemented media mimicking primordial environments, followed by 500 generations in glucose. We observed a robust and stable transition from rod-shaped to spherical morphology that persisted despite environmental reversal. This morphological shift was accompanied by mutations in cell-shape-related genes, and no reversion to the ancestral rod shape was observed during evolution in glucose, where mutations primarily affected metabolic and transcriptional pathways. These findings show that environmental pressures can drive heritable and evolutionarily stable morphological changes in bacteria. Our work provides insight into the genetic and environmental factors shaping bacterial cell geometry and is consistent with, though not conclusive for, the possibility that primordial cells may have been spherical.

Bacterial cell shape plays a fundamental role in many aspects of physiological processes, including growth, division, and spatial organization of cellular components [1,2]. This shape is primarily determined by peptidoglycan (PG) synthesis, which is spatially coordinated by the actin homolog MreB [3]. MreB filaments recruit penicillin-binding proteins to sites of lateral PG insertion, directing the cell wall remodeling required for maintaining rod-shaped morphology [4,5]. In parallel, the tubulin-like GTPase FtsZ assembles into a contractile Z-ring that orchestrates septal PG remodeling during cytokinesis [6]. Overall, bacterial cell shape emerges from the integrated activities of cytoskeletal scaffolds (e.g., MreB, FtsZ), PG biosynthesis and remodeling machineries, and diverse spatial regulators, while also dynamically responding to environmental cues, including osmotic stress and nutrient availability [7,8].

Bacterial morphological plasticity is a key survival strategy that enables cells to adapt to environmental stresses [9,10]. Under nutrient-starvation conditions, *Escherichia coli* undergoes a rod-to-filament transition that increases cellular surface area [11]. *Caulobacter crescentus* similarly adapts to nutrient-limited environments through stalk elongation, a specialized cell extension of the envelope [12]. Notably, even closely related species of the *Caulobacteraceae* employ distinct modes of cell elongation, highlighting a remarkable degree of phenotypic plasticity within this group [13]. Beyond morphological plasticity, bacteria achieve long-term adaptation through evolutionary processes. When exposed to persistent selective pressures, spontaneously arising beneficial mutations can become enriched in a population, ultimately reaching fixation if they confer sufficient fitness advantages [14]. Through this process, transient phenotypic responses can be transformed into heritable genomic traits, allowing bacterial lineages to stabilize adaptive functions in challenging environments. Experimental evolution studies have further demonstrated that even subtle environmental pressures can drive substantial phenotypic and genotypic changes over time, including long-term alterations in cell morphology [15,16]. For example, *Escherichia coli* adapted to high-osmolarity conditions exhibit reduced growth rates accompanied by increased cell volume, illustrating how experimental evolution can illuminate the complex interactions between cellular networks and environmental constraints [17]. In addition, bacterial cell size homeostasis is fundamentally governed by fatty acid availability, which directly limits cell envelope biosynthesis and thereby determines cell dimensions through membrane-limited expansion. This conserved mechanism supersedes earlier models of size regulation and establishes lipid metabolism as a primary driver of morphological adaptation [18,19].

The compartmentalization of functional molecules within discrete, membrane-bound structures is widely considered essential for the origin, persistence, and evolution of living systems [20]. In this context, the protocell is conceptualized as a primitive, membrane-enclosed entity capable of supporting the replication of early genetic materials and facilitating rudimentary catalytic processes [21,22]. Fatty acids have garnered particular attention as plausible prebiotic membrane constituents due to their simple chemical structures and intrinsic capability to self-assemble into vesicular compartments under prebiotically relevant conditions [23]. Among them, oleic acid (C18:1), a monounsaturated fatty acid, has been extensively investigated as a model component of primitive cellular membranes [24–26]. In a prior study employing oleic acid vesicles (OAVs) as a mimic for primordial environments, experimental evolution of *E. coli* over approximately 500 generations resulted in enhanced bacterial fitness accompanied by a morphological transition from rod to sphere [27]. Genomic analyses revealed two distinct adaptive strategies, either directly targeting the cell wall or operating through alternative pathways. These findings offer empirical support for the notion that spherical morphology may have been selectively favored in early protocellular environments [27].

However, given the relatively limited evolutionary timescale (∼500 generations), it remains unclear whether the observed morphological adaptations are transient responses or stable, heritable traits. First, it is unclear whether ∼500 generations constitute an insufficient duration to capture the full spectrum of morphological diversification. Over longer evolutionary periods, additional morphological transitions may emerge. Moreover, as fitness continues to increase, the selective advantage associated with spherical morphology may diminish, potentially weakening or even eliminating this shape adaptation. Second, because OAVs impose a strong selective pressure favoring the spherical form, an important question is whether reintroducing glucose as the primary carbon source would reverse this adaptation—restoring the canonical rod shape—or instead promote the emergence of alternative phenotypes. Third, our prior research pinpointed both direct and indirect genetic mutations during the initial 500 generations [27]. It remains unknown whether extended evolution in the OAV-rich environment would lead to the accumulation of additional mutations directly regulating cell shape. Upon shifting the carbon source from OAVs to glucose, it is essential to determine which mutations ultimately reach fixation in the population and to elucidate their functional consequences and fitness impacts.

In the present study, we extend the experimental evolution of six previously derived *E. coli* lines (Evos) for an additional ∼500 generations in OAVs or glucose. When propagated for another 500 generations in the OAV-rich environment, all six lineages retained the spherical morphology, with no evidence of reversion to the ancestral rod shape, and their growth rates exhibited clear stabilization. Whole-genome resequencing of these ∼1000-generation lineages revealed that five of the six accumulated mutations in genes directly implicated in cell morphology—an increase relative to the initial 500-generation interval, during which only three lineages acquired such direct morphological mutations. When the same six lineages were evolved for 500 generations in glucose, the spherical morphology likewise persisted. No lineage reverted to a rod-shaped state or displayed alternative morphological transitions, indicating that the spherical phenotype is stably maintained and is not reversed by changes in carbon source. Evolution in glucose was characterized by a greater prevalence of mutations associated with transcriptional and translational regulation, broadly influencing cellular growth and metabolic processes. By contrast, evolution in the OAVs environment preferentially enriched mutations linked to determinants of cell morphology. Through systematic analysis of evolutionary trajectories in both morphology and fitness, this work seeks to clarify the stability of shape evolution and to provide deeper insight into how selective pressures imposed by OAVs may have influenced the morphological characteristics of early cellular life.

## Materials and methods

### *E. coli* strain

The laboratory-evolved strains, L# (L31, 32, 9–12), were evolved in OAV-supplemented minimal media for 500 generations [27]. The original strain was *MDS42ΔgalK::P_tet_-gfp-kan* named Ori, which was derived from the wild-type genome MG1655 by removing transposons [28,29].

### Media

The OAV-supplemented minimal media were prepared as described previously with minor changes [30]. In brief, the media were prepared by mixing three stock solutions of base salts, trace elements, and OAVs with ddH_2_O to a final concentration of 62 mM K_2_HPO_4_, 39 mM KH_2_PO_4_, 15 mM (NH_4_)_2_SO_4_, 0.009 mM FeSO_4_, 0.015 mM thiamine hydrochloride, 0.2 mM MgSO_4_ and 3.5 mM OAVs. The glucose-supplemented minimal media were identical to that of the OAVs medium, except for the replacement of 3.5 mM OAV with 10.5 mM glucose.

### Experimental evolution

Six independent evolutionary lineages evolved in either OAVs (Ln) or glucose (LnG, L31G, 32G, 9G–12G) were generated from L# (L31, 32, 9–12) as described above. The cells were cultured in 3 ml of a minimal medium with 3.5 mM OAVs or 10.5 mM glucose, and the cell cultures were incubated in a bioshaker (MBR-022UP, Taitec, Japan) with a rotation rate of 200 rpm at 37°C. For daily serial transfers, the growth stage of each culture was estimated by measuring the optical density at 600 nm (OD_600_) using a microplate reader (Synergy H1, BioTek, U.S.A.). The 96-well plates were incubated in the plate reader with continuous orbital shaking at 282 cpm and 37°C. To ensure that the transfers occurred during the exponential phase, despite minor lineage-specific differences in growth rates, three dilution ratios were applied at each transfer. The OD_600_ values were averaged across three technical replicates. The growth rate was determined as the change in OD_600_ between the beginning and the end of 24-h cultivation period, normalized by the cultivation time. Among the three replicate cultures, only the culture that remained in exponential growth was selected for subsequent serial transfer. The six lineages were generated independently to avoid cross-contamination. The daily cell cultures were all stocked with 15% glycerol at –80°C.

### Imaging flow cytometry

The *E. coli* cell populations were analyzed using an Amnis™ ImageStream™X imaging flow cytometer installed with INSPIRE acquisition software (Luminex, U.S.A.) as described previously [27]. After completion of the full evolutionary experiment, the frozen samples collected at approximately 50-generation intervals were revived in the corresponding media and subsequently analyzed by imaging flow cytometry (IFC). Cell morphology within each population was quantified using the aspect ratio, as defined in our earlier work [27]. Briefly, the aspect ratio was calculated as the ratio of the cell’s minor axis length to its major axis length, corresponding to the narrowest dimension divided by the longest dimension of each captured cell image.

### Confocal microscopy

All image acquisition of the *E. coli* cells was processed by confocal microscopy (C2 plus, Nikon, Japan). The bacteria were collected by centrifugation for 1 min at 5000 × ***g*** and then 1.5 μl of bacterial suspension was placed on a glass slide. The samples were observed on a 100× magnification oil lens (Nikon).

### Scanning electron microscopy

The *E. coli* cells were fixed with 2.5% glutaraldehyde and further treated with 1% OsO_4_ for 1 h at 4°C. Cells were rinsed with phosphate-buffered saline three times, dehydrated in a graded series (30%, 50%, 70%, 80%, 90%, 95%, and 100%) of ethanol, dried with a critical point dryer (Leica EM CPD 300, Leica Microsystems GmbH, Wetzlar, Germany), and coated with gold in a sputter coater (ACE600, Leica Microsystems). The prepared samples were observed using a scanning electron microscope (SEM) (Hitachi S-4800, Japan) at an accelerating voltage of 3 kV.

### Genome mutation analysis

The evolved strains grown in glucose-supplemented minimal media were harvested at the stationary phase for genome mutation analysis, as described previously. Genome sequencing was performed by Sangon (Shanghai, China). Genomic DNA was extracted by a Magen Bacterial DNA KF Kit (Sangon, Shanghai, China), and gDNA libraries were constructed using the HieffNGS MaxUp II DNA Library Prep Kit for Illumina (NEB, U.S.A.). Whole-genome sequencing was performed with the HiSeq 2500 (Illumina, San Diego, CA) according to the manufacturer’s instructions. Reads were mapped to the reference sequence (NCBI accession number NC_020518.1), and the genome mutations, i.e., SNPs and InDels, were determined with the Genome Analysis Toolkit (GATK). All the mutations were analyzed and confirmed using *breseq* (v0.39.0) [31]. Raw sequencing data have been deposited at BioProject under accession number PRJNA1265462 (SAMN48632894–SAMN48632905).

### Statistical analysis

Statistical analyses and curve fitting were performed using GraphPad Prism (ver. 8.0). Nonlinear logarithmic regression was applied for curve fitting. One-way analysis of variance (one-way ANOVA) was used to assess statistical differences among lineages, and the corresponding *P*-values were calculated. A *P*-value <0.05 was considered statistically significant.

## Results and discussion

### Experimental evolution of *E. coli* in OAV or glucose-supplemented conditions

**Figure 1:**
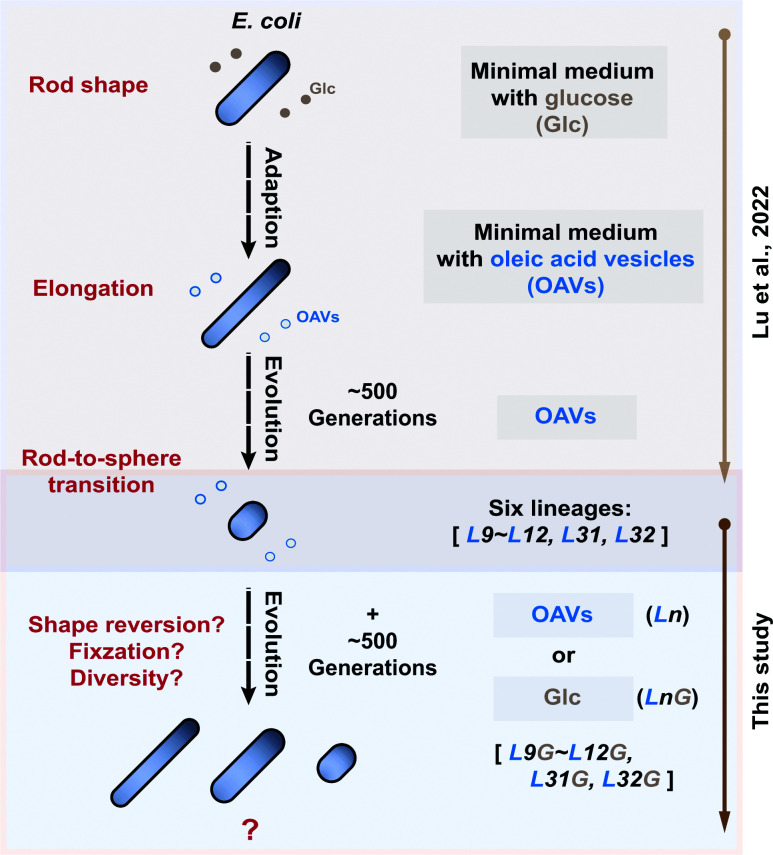
Schematic diagram of the experimental evolution of *Escherichia coli* in a primordial environment Our previous work suggested that the primitive spherical form in *Escherichia coli* evolved under primordial-like conditions [27]. In the present study, we extended the experimental evolution of *E. coli* for ∼1000 generations in OAV-supplemented media, followed by 500 generations in glucose. The fitness, cell morphology, and genomic mutations in all lineages were monitored simultaneously and investigated.

Previous work in our laboratory showed that when *E. coli* populations evolved for 500 generations in OAV-supplemented media—mimicking the resource regime of primordial environments—the cell shape shifted from a rod-shaped to spherical morphology. In contrast, populations evolved in parallel for 500 generations in glucose-supplemented media retained their typical rod shape [27]. While these findings demonstrate that OAVs can induce cell rounding within 500 generations, it remains unclear whether this morphological shift is stably maintained after removal of the selective pressure or represents only a transient physiological response. To address this question, we evolved the six previously obtained OAV-adapted strains (Evos: L31, L32, and L9–12) for an additional 500 generations under two parallel conditions (Figure 1): continued evolution in OAVs, to assess whether cells further increase in sphericity or reach a morphological plateau; and reversion in glucose-supplemented medium (LnG), to evaluate whether the spherical phenotype is reversible in the absence of OAV selection. Serial transfers were performed to maintain cultures in the exponential growth phase, and cell density was monitored daily by measuring OD_600_ with a microplate reader. Cell morphology was quantified every 50 generations using IFC. Importantly, this glucose-reversion experiment differs from the initial glucose-based evolution performed previously [27]. Here, the aim was to examine the phenotypic plasticity of OAV-adapted strains rather than to re-evaluate ancestral glucose adaptation. To monitor potential morphological reversion in the absence of OAVs, all six Evos lineages were also cultivated in parallel for approximately 500 generations under glucose-supplemented conditions (LnG), the preferred carbon source for *E. coli* (Figure 1). As also depicted in Figure 1, it is worth noting that the data related to all six OAV-adapted lineages up to 500 generations were adopted from our earlier published results [27].

### The fitness increased under supplementation with either OAVs or glucose

**Figure 2:**
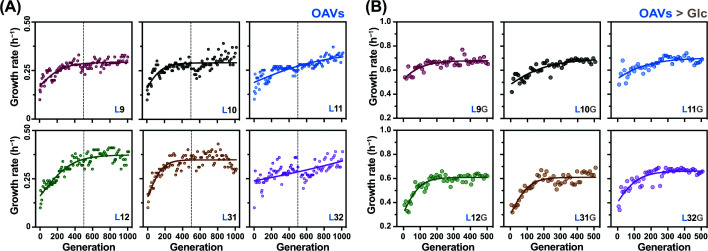
Temporal changes in the growth rate of *E. coli* (**A**) Changes in the growth rate in OAV-supplemented conditions. The subsequent serial transfer utilized a single culture from the three varying dilutions. The growth rate of the selected culture was used to assess temporal changes over 1000 generations. Logarithmic regression of the temporal changes is represented by the solid curve. (**B**) Changes in the growth rate in glucose. Six lineages evolved for 500 generations in OAVs were subsequently evolved in glucose for 500 generations. Logarithmic regression of the temporal changes is represented by the solid curve.

Six lineages evolved in the OAV-supplemented media for approximately 1000 generations. These populations exhibited rapid fitness increases during the first ∼500 generations, followed by relatively stable fitness levels over the subsequent ∼500 generations (Figure 2A). This pattern is consistent with fundamental principles of natural selection, in which beneficial mutations accumulate until population fitness approaches a plateau [32,33]. A few lineages continued to show gradual fitness improvement, likely driven by newly arising mutations that conferred additional advantages for growth in OAVs (Figure 2A). When the six OAV-adapted lineages were returned to the original glucose environment and evolved for approximately 500 generations, all lineages exhibited rapid increases in fitness (Figure 2B). This result indicates that although the bacteria had specialized for OAV utilization over 500 generations, their capability for glucose metabolism remained intact. It further suggests that no deleterious mutations affecting glucose uptake or metabolism accumulated during the earlier OAV-based experimental evolution in these lineages (Lx).

### Conserved changes in cell shape evolved in OAVs or glucose environment

**Figure 3:**
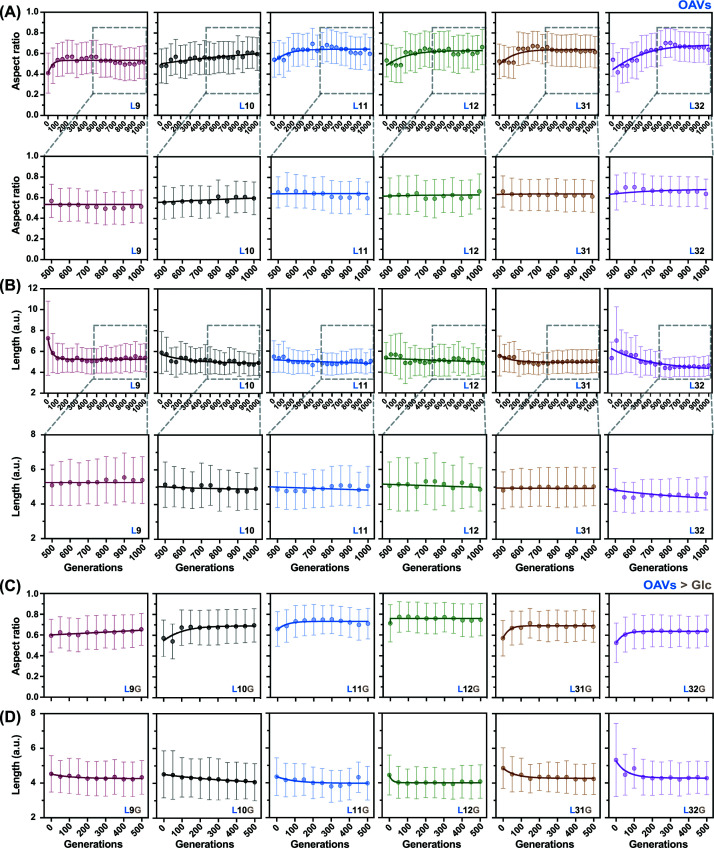
Temporal changes in cell shape and length The cell shape, represented by the mean aspect ratio, during evolution in OAVs (**A**) or glucose (**C**) is shown. Corresponding shifts in cell length are shown for OAVs (**B**) and glucose (**D**). Data points represent the mean values derived from 10 000 cells analyzed via imaging flow cytometry (IFC), with vertical bars indicating the standard deviation. Solid lines represent logarithmic regression models of the temporal trends. The labels of the six lineages evolved in OAVs (Ln) and glucose (LnG) are provided in the respective panels.

**Figure 4:**
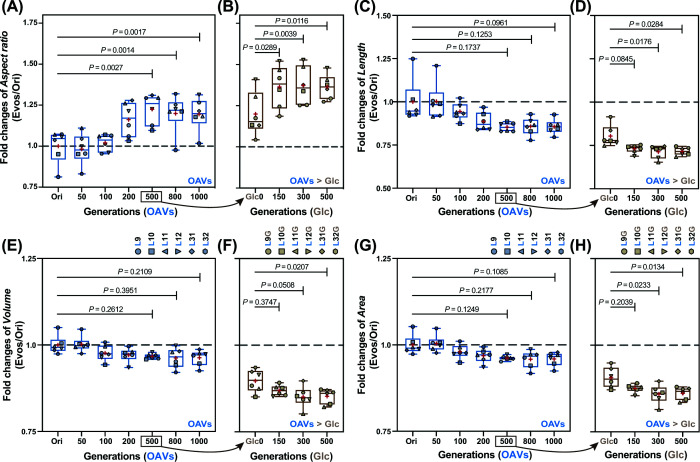
Fold changes in cell shape mediated by experimental evolution The fold changes in cell aspect ratio of lineages evolved in OAVs (**A**) and glucose (**B**) are shown in the left and right panels, respectively. The fold changes in cell length (**C,D**), cell volume (**E,F**), and cell surface area (**G,H**) under the two conditions are also presented. The fold changes were calculated as the ratios among the Ori, middle, and endpoint populations (Ln and LnG) and the boxplots indicate the distributions of the fold changes, in which the six lineages are indicated with open circles. Blue indicates evolution in OAVs, and brown indicates evolution in glucose. The red cross represents the median of the calculations. The statistical significance is indicated with the *P*-values.

The cell length and aspect ratio were measured by IFC to evaluate morphological changes. These parameters were quantified for the ancestral strain (Ori) and six evolved lineages at 100-generation intervals over a total of 1000 generations during exponential growth. Extended evolution in OAVs (∼1000 generations) further reinforced spherical morphology in all six lineages (Figure 3A,B). In addition, the fold changes in aspect ratio and cell length relative to Ori showed significant differences starting at ∼500 generations (Figure 4A,C). This indicates that a beneficial shift toward shorter (as in short rods) and spherical morphology emerged early during experimental evolution, with shape-altering mutations arising in the populations but not yet fully fixed. Consistent with this interpretation, marked spherical transformation was already detectable by ∼300 generations, indicating rapid phenotypic adaptation preceding full genetic stabilization. Additionally, fold changes in cell volume and surface area showed no significant differences before and after evolution in OAVs (Figure 4E–G).

To assess the stability of this morphological change, the six OAV-adapted lineages were further evolved for 500 generations in glucose-supplemented media. Notably, upon switching to glucose, none of the lineages reverted to a rod shape (Figure 3C,D). Instead, sphericity became more pronounced, indicating that the spherical phenotype had become environmentally independent and genetically fixed (Figure 4B–D). These findings demonstrate that *E. coli* evolved in the OAVs undergo a robust and consistent transition from rod-shaped to spherical cells. Interestingly, upon glucose reversion, the OAV-evolved lineages displayed a significant reduction in cell size and volume over the 500 generations (Figure 4F–H). This observation contrasts with previous long-term evolution studies [16,33]. This discrepancy may be attributed to several factors: (i) in the present study, the strains first evolved for 500 generations in OAV-supplemented media before glucose exposure, rather than evolving directly from the ancestral state in glucose; (ii) the glucose concentration used in the present study differs from that used in earlier studies; and (iii) 500 generations in glucose represent a relatively short evolutionary timescale compared with canonical long-term evolution experiments.

### Morphological characterization of evolved lineages

**Figure 5:**
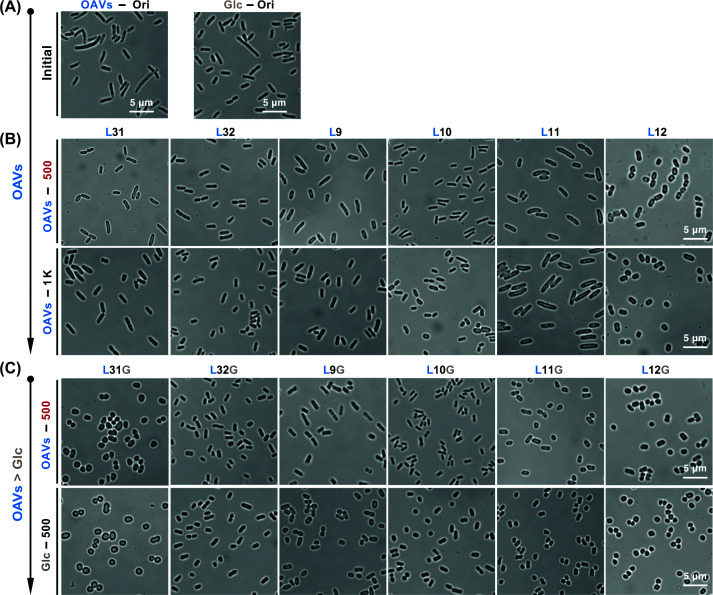
Cell shapes imaged by confocal microscope (**A**) Single-cell images of the Ori in both glucose and OAVs. (**B**) The six lineages evolved in OAVs at ∼500 and ∼1000 generations cultured in OAVs. (**C**) The six lineages initially evolved within OAVs over approximately 500 generations, followed by evolution in glucose cultures for roughly 500 generations. Scale bar = 5 μm.

**Figure 6:**
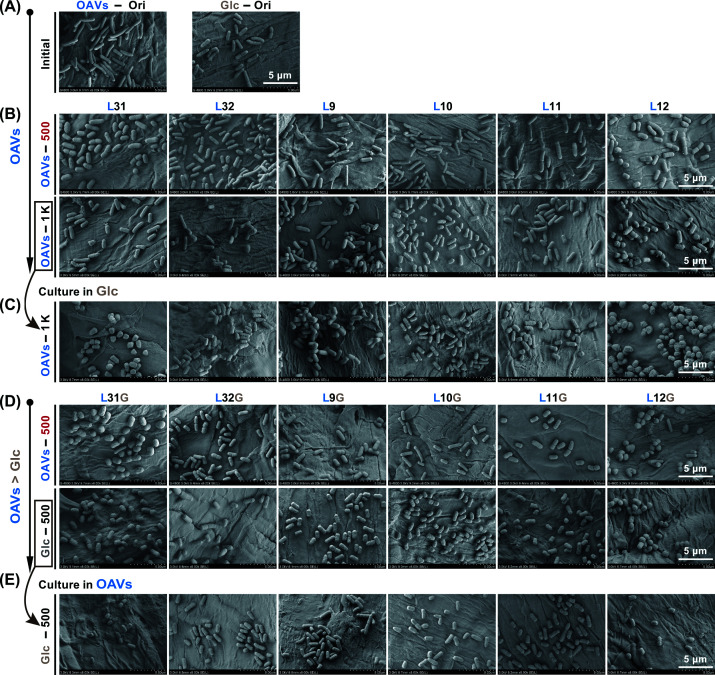
Cell shapes imaged by SEM (**A**) Single-cell images of the Ori grown in OAVs or glucose. (**B**) The six lineages evolved in OAVs at ∼500 and ∼1000 generations grown in OAVs. (**C**) The six lineages evolved in OAVs at ∼1000 generations cultured in glucose. (**D**) The six lineages initially evolved within OAVs over approximately 500 generations, followed by evolution in glucose cultures for roughly 500 generations. (**E**) The six lineages evolved in glucose at ∼500 generations cultured in OAVs. Scale bar = 5 μm.

To investigate the morphological shifts in a more detailed manner, cells in stationary phase from Ori and six evolved lineages at ∼500 and ∼1000 generations in OAVs were imaged using confocal microscopy, alongside the six Evos evolved in glucose. Cells evolved for both 500 and 1000 generations in OAVs appeared more spherical than Ori in the images, regardless of the medium in which they were imaged (Figure 5A,B). Live-cell morphology was visualized by confocal microscopy and SEM was further employed to characterize morphological differences across evolutionary stages. To more clearly compare the changes in morphology, cells evolved in OAVs at 500 and 1000 generations (Figure 6B,C) and cells evolved in glucose at 500 generations (Figure 6D,E) were cultured in both glucose- and OAVs-containing media prior to imaging. Cells cultured in OAVs consistently exhibited pronounced sphericity, consistent with the confocal microscopy observations. Moreover, all lineages that had undergone 500 generations of evolution in OAVs retained their spherical morphology after being transferred back to glucose and evolved for an additional 500 generations (Figures 5C and 6C). These findings indicate that the spherical morphology is stably maintained and does not revert when the bacteria are returned to glucose under the conditions of our experimental design.

### Divergent mutational strategies under OAVs versus glucose

**Figure 7:**
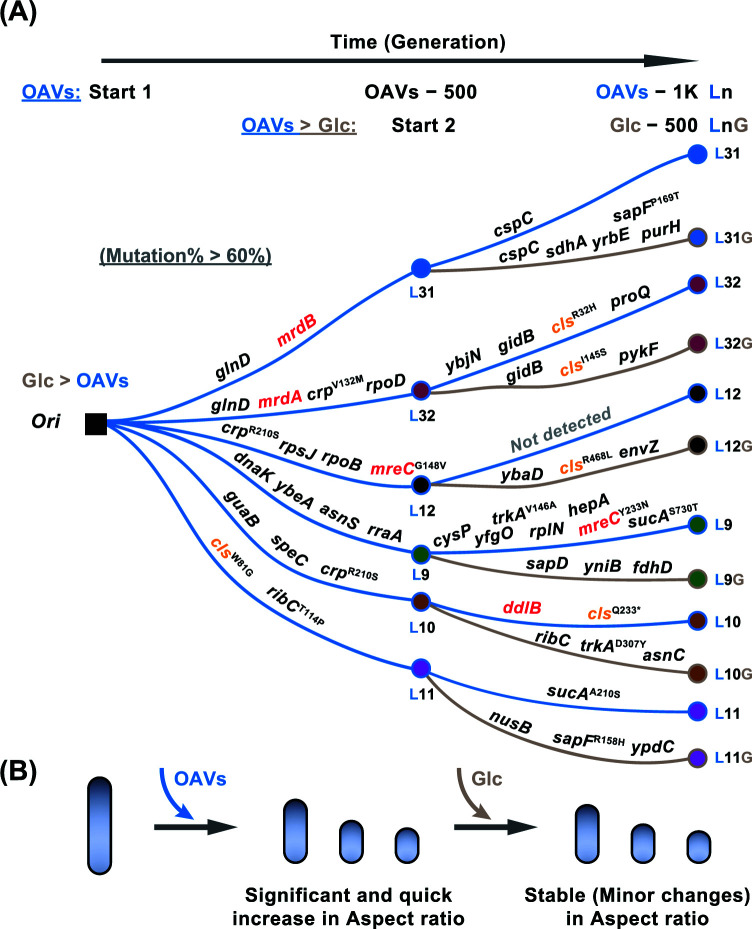
Differentiated genetic strategies (**A**) Genome mutations detected in the six lineages evolved in OAVs at 500 and 1000 generations and evolved in glucose at 500 generations are indicated. The genes that played a role in the cellular structure are highlighted in red. The mutated genes that appeared in multiple lineages are also indicated with their specific amino acid mutation sites. (**B**) The representative cell shapes of the ancestral strain and strains evolved in OAVs or glucose are illustrated.

To identify the genetic determinants underlying the observed morphological changes, we performed whole-genome sequencing on six evolved lineages at ∼500 and ∼1000 generations in OAVs, as well as on lineages evolved in glucose for ∼500 generations (Supplementary Table S1). Sequencing revealed heterogeneous mutational landscapes involving both direct and indirect evolutionary strategies. Notably, prolonged evolution in OAVs drove convergence toward direct shape-altering mutations in lineages L9 and L10 (Figure 7A). In contrast, lineages that continued evolving in glucose retained their spherical morphology but accumulated no additional mutations associated with cell shape. Together, these findings indicate that evolution in the primordial-like OAV environment promotes a robust transition from rod-shaped to spherical cells. A plausible interpretation is that the genetically entrenched spherical morphology—combined with the absence of reversion to rod shape—might provide experimental support for hypotheses suggesting early cellular sphericity in primordial environments [27,34].

Among multiple lineages (L32, L10, L11, and L12G), a recurring mutation was identified in *cls*, the gene encoding cardiolipin synthase (Figure 7A). Given its known involvement in membrane curvature and stability [35,36], *cls* represents a strong candidate for driving shape adaptation. Cardiolipin enrichment increases membrane fluidity and promotes negative curvature, consistent with its preferential localization at the cell poles [37,38]. Such biomechanical effects likely facilitate spherical morphogenesis under OAV selection, paralleling lipid-mediated size and shape control observed in modern bacteria [18]. Another key recurring mutation appeared in the global regulatory gene *crp*, identified in lineages L32, L10, and L12. It has been reported that the product of *crp* regulates carbon utilization in *E. coli*, suggesting interplay between metabolic regulation and morphological adaptation [39]. Intriguingly, after ∼1000 generations, mutations associated with cell wall synthesis or cell shape appeared in five of the six lineages—an increase from three lineages in the first 500 generations (Figure 7). This pattern reflects convergent selection for structural remodeling under sustained primordial-like pressures. Mutated genes included *mrdB, mrdA, mreC*, and *ddlB*, involved in PG synthesis, cross-linking of the PG cell wall, maintenance of rod shape, and cell wall formation [40–45]. These results imply that evolution in OAVs drives a direct genetic transition from rod-shaped to spherical cells. By contrast, when the OAV-evolved lineages were subsequently evolved in glucose for 500 generations, mutations that accumulated were primarily associated with carbon metabolism or transcription and translation (Figure 7A). No known cell-shape or cell-wall-related genes were mutated. Among these, *pykF*—a gene frequently mutated in the long-term *E. coli* evolution experiments—was also observed [46].

In our previous research, we provided the first experimental evidence of rod-shaped bacterial cells changing to spherical shapes in a laboratory environment mimicking the resource regime of the primordial environment [27]. Nevertheless, within the relatively short evolutionary timeframe examined, it remained unclear whether these morphological changes were heritable or merely transient physiological responses. We initially speculated that spherical morphology—by reducing the surface-area-to-volume ratio—could help mitigate osmotic stress in fatty-acid-rich environments. In the present study, we extended the evolution of six lineages in OAVs to 1000 generations and confirmed that the spherical morphology is stable and persistent. Notably, mutations in cell-shape-determining genes emerged in five of the six lineages, indicating that OAV-mediated selection promotes both the emergence and long-term maintenance of spherical morphology (Figures 3-7). Consistent with our earlier hypothesis, reduced surface-area-to-volume ratios in spherical cells [47] may indeed lessen osmotic pressure in fatty-acid-rich conditions. Furthermore, when OAV-adapted bacteria at 500 generations were re-evolved to their ancestral carbon source (glucose) for an additional 500 generations, they persisted as spheres without reverting to the ancestral rod shape. Crucially, these glucose-evolved populations accumulated no novel mutations related to cell shape; instead, mutations primarily affected transcription- and translation-associated pathways, revealing divergent evolutionary trajectories under distinct nutrient constraints. The persistence of sphericity in glucose—despite the absence of further shape-related mutations—argues against the assumption about phenotypic plasticity. We proposed that the mutations disrupting rod-maintaining machinery (e.g., *mreB* cytoskeleton; Figures 6 and 7) may be functionally irreversible. In *E. coli*, deletion of *mreB* induces spherical morphogenesis but is lethal upon reintroduction, suggesting strong structural constraints on reversion. Additionally, shape-altering mutations such as those in *cls* (cardiolipin synthase; Figure 7A) may enhance membrane stability under OAV conditions but inadvertently stabilize sphericity even in glucose.

Across the full course of evolution in OAVs, bacterial populations underwent a robust and persistent transition to spherical morphology, accompanied by extensive genomic diversification. Shape-determining mutations arose in five of six lineages, with fixation occurring during both early (0–500 generations) and later (500–1000 generations) phases. This convergent genetic targeting underscores both the heritability and adaptive stability of the spherical phenotype. Although further mechanistic validation is needed and the evolutionary timescale remains shorter than canonical long-term evolution studies, the rapid fixation of spherical morphotypes suggests that OAVs strongly promote convergent morphological evolution.

## Limitations and perspectives

The present study investigated the experimental evolution of *E. coli* under OAV conditions and identified a stable rod-to-sphere transition. While these results provide an informative starting point, several limitations remain.

(i) Although whole-genome sequencing revealed mutations in multiple cell-shape-related and metabolism-associated genes, their functional roles are not yet resolved. In particular, how carbon-flux reorganization differs between oleic acid and glucose conditions—and whether these metabolic shifts directly drive morphological change—remains unknown without targeted metabolic measurements. (ii) Several identified mutations could be linked to pathways related to PG biosynthesis or cell-wall regulation, yet these connections remain hypothetical. Experimental validation will be essential to determine whether these mutations mechanistically contribute to the loss of rod shape. (iii) The possibility that altered membrane lipid composition—such as changes in cardiolipin abundance or acyl-chain remodeling—affects membrane curvature and stabilizes the spherical morphology has not been tested. Lipidomics and perturbation experiments are needed to evaluate these membrane-level effects.

Beyond these limitations, this work points toward several promising research directions. (i) Different fatty acids vary in chain length, saturation, and their influence on membrane properties. Extending evolution experiments to additional fatty acids could clarify whether the observed spherical morphology is specific to oleic acid or reflects a broader lipid-driven adaptive strategy. (ii) Applying similar evolutionary frameworks to other bacterial models, such as *Bacillus* with its thick PG layer or *Lactococcus* with distinct metabolic capabilities, may help determine whether fatty-acid-induced shape evolution is conserved across taxa. (iii) Finally, combining genetic validation, metabolic flux analysis, cell-wall profiling, and membrane lipidomics will enable a more complete mechanistic understanding of how mutations, metabolism, and membrane organization interact to shape evolutionary changes in bacterial morphology.

Together, these limitations and perspectives outline the next steps needed to establish a causal and integrative model of how *E. coli* and other bacteria adapt their morphology in response to fatty-acid-based environments.

## Supplementary Material

Supplementary Table S1

## Data Availability

Genome sequencing data are deposited at BioProject with accession number PRJNA1265462 [48]. All data are available from the corresponding author upon reasonable request.

## Abbreviations

IFC: imaging flow cytometry
OAV: oleic acid vesicle
PG: peptidoglycan
SEM: scanning electron microscope
